# Linking experiences of child sexual abuse to adult sexual intimate partner violence: the role of borderline personality features, maladaptive cognitive emotion regulation, and dissociation

**DOI:** 10.1186/s40479-021-00150-0

**Published:** 2021-04-01

**Authors:** Annegret Krause-Utz, Tara Dierick, Tobias Josef, Elianne Chatzaki, Andries Willem, Jan Hoogenboom, Bernet Elzinga

**Affiliations:** 1 Institute of Psychology, Department of Clinical Psychology, Leiden University, Leiden, The Netherlands; 2 Leiden Institute for Brain and Cognition, Leiden University, Leiden, The Netherlands

**Keywords:** Borderline personality disorder, Child sexual abuse, Dissociation, Intimate partner violence, Sexual intimate partner violence

## Abstract

**Background:**

Child sexual abuse (CSA) has been linked to a higher risk of sexual re-victimization, including sexual intimate partner violence (IPV). The aim of this study was to investigate whether borderline personality disorder (BPD) features, dissociation, and maladaptive cognitive emotion regulation mediate the link between self-reported CSA severity and sexual IPV. Specifically, we were interested in the unique effect of each mediator variable, when accounting for the effect of the other variables.

**Methods:**

Data was assessed in a cross-sectional anonymous online survey, posted on platforms for people affected by domestic violence, and research platforms of Leiden University. Overall, *n* = 633 participants completed the survey (including *n* = 100 participants with CSA and *n* = 345 reporting at least one incidence of sexual IPV). Multivariate regression analyses and path-analytical modelling were performed for hypothesis testing.

**Results:**

Adult sexual IPV was predicted by more severe CSA, more severe BPD features, higher dissociation, and more maladaptive emotion regulation. Each mediator variable showed a significant effect in the separate mediation models. In the overall model, only dissociation and maladaptive emotion regulation, but not BPD features, mediated the association between CSA and sexual IPV.

**Conclusions:**

Findings add to the existing literature, suggesting that CSA severity, BPD features, dissociation, and maladaptive emotion regulation are important risk factors for sexual IPV. Given the cross-sectional correlational design of our study, prospective studies are needed to corroborate our findings regarding potential psychological mechanisms underlying sexual re-victimization. Ultimately, this can help developing interventions aimed at breaking the cycle of abuse.

**Supplementary Information:**

The online version contains supplementary material available at 10.1186/s40479-021-00150-0.

## Introduction

Child sexual abuse (CSA) can have devastating consequences across the life-span [1–9]. It occurs worldwide in various settings and entails a wide range of non-consensual, coerced sexual activity, which the child does not fully comprehend or is not developmentally prepared for (e.g., exposure to sexual organs and/or content, forced sexual acts). An estimated percentage of 19.2–19.7% women and 7.4–7.9% men have experienced sexual abuse (before age 18) [10].

Experiences of CSA increase the risk of later sexual re-victimization [1, 2, 11–13]. Sexual re-victimization may involve exploitation and assaults by strangers and/or casual acquaintances, but can also occur in romantic relationships, i.e., as repeated non-consensual or forced sexual activity by a stable relationship partner [1, 2, 11]. Within romantic relationships, sexual re-victimization often has a more chronic and pervasive nature, and is often accompanied by other forms of intimate partner violence (IPV, e.g., psychological / physical aggression) [1, 2, 14, 15]. Therefore, this form of sexual re-victimization has been studied separately with regards to possible risk factors and underlying mechanisms (see) [2].

In general, both external (societal, cultural) and intra-individual (psychological) risk factors have been implicated in sexual re-victimization [16]. In a recent systematic review by Scoglio and colleagues (2021) [1], overall severity of child maltreatment (abuse, neglect), risky sexual behavior particularly in adolescence, emotion dysregulation (e.g., maladaptive emotional coping), and post-traumatic stress symptoms were identified as most consistent risk factors for re-victimization after child abuse [1]. However, most studies included only college-age women (≤24 years), which limits the generalizability of findings for male and older populations [1]. Moreover, only few studies examined more than one mediator variable in the same model [17], while mediator variables may also affect one another [18].

With regard to sexual IPV specifically, it has been proposed that CSA, emotion dysregulation, and dissociation are key risk factor for re-victimization in romantic relationships. Trauma models propose that CSA leads to alterations in emotional and cognitive processing, which make sexually abused individuals more vulnerable to sexual revictimization in intimate relationships [2].

Emotion dysregulation is a common consequence of CSA [4]. Survivors of CSA often internalize their stressful experiences, e.g., through self-blame [12, 19], and use avoidance coping to regulate emotions in the short-run [20–23]. In the long-run, these emotion regulation strategies hinder goal-oriented problem-solving (e.g., reaching out for practical and social support) and increase emotional distress [2, 4, 12, 24]. Self-blame has been associated with delayed disclosures of CSA [25] and increased depressive symptoms and suicidal ideation at a later time point [26]. Several studies have provided evidence for a mediating effect of maladaptive emotion regulation strategies in the link between child abuse and dating violence, although contrary findings were also reported [2].

Next to maladaptive emotion regulation, dissociation is a common consequence of CSA [27], which may enhance vulnerability for sexual re-victimization [2]. Dissociative symptoms, such as depersonalization and derealization, may be understood as a form of emotion modulation, which helps creating an inner distance to overwhelming experiences, e.g., during CSA [28, 29].

Dissociation may also increase the risk of sexual re-victimization. A hypothesized reason for this association is that dissociation can lead to disturbed encoding and processing of threat-related information [28, 30, 31], i.e., poor risk recognition, and thereby interfere with safety judgements in social situations. This may in turn facilitate problematic sexual behaviours, such as exposing oneself to abusive situations and ignoring or minimizing alarm signals due to a reduced awareness of threat [31]. Dissociation may further interfere with the awareness and communication of needs and boundaries, hindering resistance against coerced sexual activity.

Findings from two prospective studies provided first evidence for the idea that dissociation is an important underlying mechanism of sexual-revictimization after child abuse. Noll, Horowitz, Bonanno, Trickett, and Putnam from 2003 [32] investigated associations between dissociation and lifetime trauma histories in females with confirmed histories of CSA. Abused participants reported significantly more domestic violence and subsequent lifetime traumas than the comparison group. Sexual revictimization was positively correlated to dissociation. In another prospective study by Zamir and colleagues (2018) [33], CSA predicted more dissociation in late adolescence (age 19), which in turn predicted more IPV during early to mid-adulthood (ages 20–32). Dissociation partially mediated the effect of CSA on IPV. However, more research is needed to understand the role of dissociation in the relation between CSA and sexual IPV, especially in the context of other mediator variables.

In their systematic review of the current literature, Hébert and colleagues (2020) [2] concluded that evidence in the field is still mixed, calling for more research in this area. More specifically, most studies focused on post-traumatic stress disorder (PTSD) symptoms [2], while other psychopathologies that are linked to CSA and IPV, such as borderline personality disorder, have been researched less.

Borderline personality disorder (BPD) is associated with more dating violence in adolescence [34] and IPV in adulthood [19, 35–38]. Even though a history of trauma is neither necessary nor sufficient for the development of BPD [10], higher rates of CSA have been found in patients with the disorder compared to other psychiatric groups [3, 39–42]. It is assumed that an interplay of stressful experiences, such as CSA, with vulnerability factors (e.g., genetic and neurobiological imbalances) leads to the development and maintenance of BPD features [43, 44].

Emotion dysregulation, identity disturbances (including dissociation), and risky self-harming behaviour are core features of BPD [43], which were found to be more pronounced in individuals who experienced CSA [45]. As previously mentioned, these factors are also thought to increase the risk for sexual-revictimization.

First evidence for a mediating effect of BPD features on the link between child abuse and IPV stems from a previous study by Krause-Utz et al. (2018) [19]. In this study, female and male participants (*n* = 703) performed an anonymous online survey. Significant correlations between severity of child maltreatment (abuse, neglect), BPD features, maladaptive cognitive emotion regulation, and IPV were found [19]. BPD features but not maladaptive cognitive emotion regulation mediated the association between child maltreatment (emotional, physical, sexual abuse and neglect) and IPV.

The mediating effect of BPD features remained significant, when accounting for maladaptive emotional regulation as additional mediator. There was no significant mediated mediation (of BPD features via emotional regulation). Among the four core BPD features, disturbed identity and relationship problems had a unique mediating effect, when accounting for affective instability and self-harm.

Disturbed identity is closely linked to dissociation in BPD [46, 47]. This raised the question whether BPD features may still have a mediating effect, when accounting for experiences of dissociation. Since Krause-Utz et al. (2018) [19] did not include a measure of dissociation, the current study aimed to address this gap.

Overall, the current study aimed to shed more light on associations between CSA, BPD features, dissociation, and maladaptive cognitive emotion regulation, and sexual IPV. As it has been proposed that CSA and its sequelae (including dissociation) are a key factor for sexual re-victimization, specifically occurring in romantic relationships [2], we focused on this form of IPV in the current study. More specifically, we investigated whether BPD features, dissociation, and maladaptive cognitive emotion regulation constitute potential psychological pathways through which CSA increases the risk of experiencing sexual IPV.

Using an anonymous online survey, we expected to replicate earlier findings that CSA, BPD features, maladaptive cognitive emotion regulation, and dissociation positively predict the frequency of sexual IPV (hypothesis 1). We further hypothesized that maladaptive emotion regulation, dissociation, and BPD features are significant mediators in the link between CSA and sexual re-victimization (IPV) (hypothesis 2). Additionally, we explored the unique effects of the above-mentioned mediators, when including all mediators in one model. Following up on a previous study [19], we expected that dissociation would partly mediate the mediating effect of BPD features.

## Methods

### Participants

Data collection took place at the Faculty of Social and Behavioural Sciences of Leiden University in intervals between March 2018 and April 2020. Participants were recruited via online platforms directed at individuals who experienced violence in childhood and/or adulthood (administrators gave permission to post our survey on that platform) as well as general social media (e.g., Facebook, Twitter) and the research participation site of Leiden University. Inclusion criteria, as indicated in the information letter at the very beginning of the survey, were providing informed consent, being at least 18 years old, having sufficient English proficiency, and having had a long-term relationship. Overall, *n* = 1029 participants opened the online survey, *n* = 60 indicated that they did not have sufficient English proficiency or that they have not had a long-term relationship and therefore could not proceed with the survey, which was then automatically terminated. From the remaining *n* = 969 respondents, *n* = 643 (66%) completed all relevant scales; *n* = 10 had to be excluded because they terminate the survey before completing all necessary scales; *n* = 5 of them indicated that they did not understand all questions due to a lack of English proficiency. Demographic information for this final sample (*n* = 633) and a comparison with the initial sample can be found in Supplemental Table 1.

In the final sample, most participants were female (*n* = 448, 70.8%), European (*n* = 528, 83.4%), currently in a relationship (*n* = 342, 54%, with *n* = 89 (14%) being married), and had secondary school education (*n* = 343, 54%).

### Material

#### Childhood trauma questionnaire – short form (CTQ-SF)

The CTQ-SF measures self-reported sexual abuse and other forms of abuse and neglect in childhood [48]. Twenty-eight items are answered on a five-point Likert scale ranging from (1) never true to (5) very often true. Five subscales measure emotional abuse, physical abuse, sexual abuse, emotional neglect, and physical neglect. For the current study, the subscale on childhood sexual abuse was the primary outcome measure, while the other subscales were of secondary interest. Higher sum scores on each scale represent higher severity of abuse. The CTQ previously showed convergent validity with therapist ratings, good test-retest reliability (ranging from .79 to .84) and internal consistency coefficients between *α* = .66 and .92 [48]. Good psychometric properties were also found for the short form [49, 50]. In the current study, internal consistency (Cronbach’s alpha) was very good (sexual abuse subscale: *α* = .937; emotional abuse: *α* = .873, physical abuse: *α* = 943; emotional neglect: *α* = .953) except for physical neglect (*α* = .597). Combining the two subscales on emotional and physical neglect improved internal consistency in our sample (*α* = .912).

#### Conflict tactics scale revised 2 (CTS-2)

The CTS-2 subscale ‘sexual coercion‘ [51] was used to assess sexual intimate partner violence (e.g., “my partner insisted on sex when I did not want to”, “my partner used force like hitting, holding down, to make me have sex”, “my partner used threats to make me have sex”) and their frequency within a relationship (0: this has never happened, 1: once, 2: twice, 3: 3–5 times, 4: 6–10 times, 5: 11–20 times, 6: more than 20 times). Pairs of questions are asked referring to the self (preparation) and the partner (victimization). The items on “victimization” of this subscale were summed up and used as outcome in the present study. Internal consistency was *α* = .910.

#### Personality assessment inventory – borderline feature scale (PAI-BOR)

This 24-item self-report inventory was derived from the Personality Assessment Inventory, guided by theoretical, diagnostic conceptualizations, empirical re- search, and evaluation of psychometric properties [52]. Four subscales measure the BPD features of Affective Instability, Identity Disturbance, Negative Relationships, and impulsive Self-Harm, with six items per subscale. Statements are rated on a four- point Likert scale from (0) false to (3) very true. In the current study, the score on the total scale was used as it represents the overall levels of BPD features. A raw score of 38 or higher on this total scale acts as a cut-off for BPD features that fall into the range of clinical significance. In previous research, internal consistencies ranged from .77 to .84 [53]. Internal consistency of the total scale in the present study was *α* = .71.

#### Dissociative experience scale (DES)

The DES (50) includes 28 items (e.g., “[…] finding yourself in a place and have no idea how you got there”), which are rated on a 0–100% scale from 0% (never applied to me) to 100% (always applies to me). It conceptualizes dissociation as a general tendency to experience dissociation (trait dissociation) rather than current dissociative experiences and can be applied both in clinical and non-clinical populations. Items measure different forms of dissociation (absorption, depersonalization, derealization, dissociative amnesia) [54]. An established cut-off for this scale is an overall mean score of 30. In the current study, the total DES score was used as a measure of overall levels of trait dissociation. The DES was found to have high convergent validity and internal consistency (*α* = .93) [55]. In our sample, internal consistency for this total scale was *α* = .913.

#### Cognitive emotion regulation questionnaire (CERQ)

Maladaptive cognitive emotion regulation was assessed using subscales of the 18-item short version of the CERQ [56]. Items refer to the use of cognitive emotion regulation strategies after having experienced a negative life event: (between ‘1= almost never’ to ‘5= al- most always’). For the current study, the subscales rumination (repetitive thinking about aspects and feelings associated with the event), catastrophizing (emphasizing the terror of the experience), and self-blame, which are linked to more maladaptive mental health outcomes were included [57]. All subscales previously showed good internal consistencies and reliability [57]. In the current study, internal consistency for the maladaptive scales was *α* = .886.

### Procedure

The study was approved by The Psychology Ethics Committee of Leiden University (CEP19–0307/174). Data collection took place between March 2017 and May 2020.

through an online survey using the software Qualtrics (*Q*c 2015, Qualtrics, Provo,

UT). A link and a QR code, both directing to the survey, were presented as online post at platforms for people who experienced domestic violence in childhood and/or adulthood. We additionally advertised the study on the online research participation site of Leiden University, by flyers distributed in the University building, and on social media (Twitter, Facebook). Due to the sensitive nature of the questions, we added a disclaimer to the posts and in the information letter [“Please do not participate in this survey if you are in a current crisis or very upset about certain events. Participating in this survey will likely induce emotional distress (e.g., trigger unpleasant memories, feelings, and thoughts).”]. Participants were informed about the aim and background of the study, including potential risks, reimbursement for participation, and the right to terminate the survey at any point of time without negative consequences. Access to the survey was only possible after agreeing on the informed consent and indicating that inclusion criteria were fulfilled. At the beginning of the survey, respondents were asked to provide demographic information (age, gender, education, nationality, relationship status). Afterwards the above-mentioned scales (CTQ, CTS-2 subscale, PAI-BOR, DES, CERQ) were presented in randomized order. We used a forced choice item format to prevent missing values, participants either had to complete all questionnaires or needed to terminate the survey (*n* = 10 participants made use of this option).

After completing these scales, participants were fully debriefed and asked whether they were “[…] unable to answer one or more questions due to a lack of English proficiency” (a YES response led to post-hoc exclusion from the analysis in *n* = 5 cases). Participants were explicitly encouraged to seek contact with the principal investigator (AKU), a trained clinical psychologist, in case of discomfort (*n* = 23 participants contacted the PI, but no psychological intervention was needed. The survey took 35–45 min to complete. Respondents had the opportunity to participate in a lottery (chance of winning one of 11 25 Euro Amazon vouchers). Psychology students could alternatively choose to gain study credits.

### Statistical analysis

Data was exported to and subsequently analyzed with IBM SPSS Statistics 24.0, with a-priori *α*-value of *p* ≤ 05, two-tailed. Childhood sexual abuse (CSA) was represented by the mean sum score of the CTQ subscale ‘sexual abuse‘; BPD features by the mean PAI-BOR total score; dissociation was operationalized as mean DES sum, and maladaptive cognitive emotion regulation as mean of the ‘maladaptive‘CERQ subscales. The total score on the CTS-2 subscale ‘sexual coercion‘was our outcome measure for frequency of sexual intimate partner violence (re-victimization).

Prior to the analyses, assumptions of linearity, normality of residuals, homoscedasticity and independence of residuals were checked. Deviation from normality was detected for CTQ and therefore non-parametric tests were applied. No outliers and influential data cases (Cook’s distance, Leverage values) were identified. Multicollinearity, according to VIF and tolerance values, was not a concern. We used grand mean centering of predictors to additionally reduce multicollinearity. Underlying associations between variables were tested using Spearman correlations. Significant correlations between all variables supported our conceptual mediation model described below (all *p* < .05; Supplemental Table 2). To test the first hypothesis, that severity of CSA, BPD features, dissociation, and maladaptive cognitive emotion regulation positively predict sexual IPV frequency, several multiple linear regression analyses were performed with the mean total score of the CTS-2 subscale ‘sexual coercion‘as dependent variable. In the first analysis (model 1), mean sum scores of the CTQ subscales emotional abuse, physical abuse, sexual abuse, and the mean of the subscales emotional neglect and physical neglect were included as predictor, testing if CSA was specifically related to frequency of sexual partner violence, when controlling for other forms of abuse and neglect. In the second analysis (model 2), mean PAI-BOR total score was used as the predictor of sexual IPV. Model 3 contained mean CERQ scores as predictor, while the last analysis included mean DES sum score as predictor (model 4). The last model included all variables to test their unique predictive effect (while controlling for each other’s effect) (model 5). In all analyses, age and gender were included as covariates.

To test the second hypothesis that BPD features, dissociation, and maladaptive cognitive emotion regulation styles mediate the relationship between CSA and sexual violence (re-victimization) in adult relationships, path analytical modeling was performed using the PROCESS macro for SPSS by Hayes and Preacher [58].

In each analysis, frequency of sexual abuse in intimate relationships (CTS-2 sexual coercion subscale) was defined as outcome measure (Y variable) and severity of CSA (CTQ sexual abuse subscale) was the predictor (X).

We first performed several separate simple mediation analyses. BPD features (PAI-BOR total), cognitive emotion regulation (CERQ ‘maladaptive‘subscale score), and dissociation (DES sum score) respectively were included as mediator variable. Since we were interested in the unique effect of each mediator variable, when accounting for the effects of the other mediator variables, we also performed a multiple mediation analysis in which all three mediator variables were included together in one model. Here, we also tested for mediated mediation. For all analyses, a bootstrapping function based on 5000 samples and a confidence interval of 95% was used to quantify direct and indirect effects [58]. Analyses were performed in the full sample (*n* = 633), as we were interested in dimensional relationships of all variables. To test the robustness of our findings for participants who endorsed CSA, we repeated the mediation analysis in this subsample (*n* = 100).

## Results

### Clinical characteristics

#### Full sample

Table 1 presents means, standard deviation, and range of scores on the CTQ, PAI-BOR, DES, CERQ, and CTS-2 in the group of participants completing the survey (*n* = 633). Out of these participants, *n* = 345 (54%) reported at least one incidence of sexual IPV. This relatively high prevalence is probably be due to the fact that we recruited at online platforms for sufferers from domestic violence. In the full sample (*n* = 633), *n* = 160 (25% out of *n* = 643) presented with clinically relevant BPD features, i.e., scored above the established cut-off for this scale (> 37 on the PAI-BOR total scale) [52]. This percentage is much higher than the 1.4% prevalence, observed in a large representative sample of *n* = 8527 participants [59]. Likewise, on the dissociation scale (DES), *n* = 132 (21%) scored above the cut-off score ≥ 30 [54], which is much higher than the 2% that were identified as having pathological dissociation (DES > 30) in a previous representative sample (*n* = 1007) adults [60]. The relatively high prevalence of BPD features and dissociation may again be due to our selective sampling at websites for victims of IPV.

**Table 1:** Distributions of CTQ, PAI-BOR, DES, CERQ, and CTS-2 in the full sample *(n = 633)*

Variable *N* = 643	Mean ± SD	Minimum	Maximum
*CTQ*			
Sexual abuse	5.74 ± 2.48	5.00	25.00
Emotional abuse	8.36 ± 4.09	5.00	25.00
Physical abuse	6.00 ± 2.43	5.00	25.00
Neglect	7.88 ± 2.69	4.50	25.00
*PAI-BOR* total	25.53 ± 11.73	1.00	65.00
*DES* mean	18.45 ± 14.84	1.00	85.71
*CERQ* (maladaptive) mean	4.33 ± 3.38	1.00	17.33
*Sexual IPV victimization*	6.60 ± 9.96	0.00	69.00

*Note. CTQ* Childhood Trauma Questionnaire, *CTS-2* Conflict Tactic Scale Revised, Subscale Sexual Coercion; *PAI-BOR* Personality Assessment Inventory-Borderline Features, *CERQ* Cognitive Emotion Regulation Questionnaire;

*DES* Dissociative Experience Scale, *SD* Standard Deviation

#### Subsample of participants reporting CSA

Based on established cut-offs [61], *n* = 100 participants (16%), mostly women (*n* = 88, 14%) reported sexual abuse in childhood. The higher percentage of women is in line with findings for more representative samples; a meta-analysis of these studies suggests that 19.2–19.7% of women and 7.4–7.9% of men in the general population experienced CSA [10].

In this subsample of participants endorsing CSA (*n* = 100), *n* = 46 participants (46%) reported “severe to extreme” CSA (cut-off: ≥13 on the CTQ subscale), *n* = 40 participants reported experiences categorized as “moderate to severe” (cut-off: 8–12), and the rest (*n* = 14) fell in the category “mild to moderate” (cut-off: 6–7) [61]. Participants who reported CSA also scored significantly higher on other forms of childhood maltreatment than participants who did not report CSA (emotional abuse: 12.01 ± 5.67 versus 7.90 ± 3.62; physical abuse; 7.83 ± 4.28 versus 5.85 ± 2.09; neglect: 10.15 ± 3.65 versus 7.71 ± 2.27; *Z* = 8.37, *p* < .0001).

This group reported significantly higher levels of dissociation (29.94 ± 18.66 vs. 20.17 ± 14.66; *t*_(186.22)_ = 5.69, *p* < .0001) and BPD features (29.94 ± 12.26 vs. 24.79 ± 11.49; *t*_(139.87)_ = 3.99, *p* < .0001), but did not differ in cognitive emotion regulation (4.84 ± 3.84 vs. 4.31 ± 3.84; *t*_(186.22)_ = 1.33_(135.55)_, *p* > .05).

### Predictors of sexual intimate partner violence (total effects)

Table 2 summarizes results of the multiple linear regression analyses predicting sexual IPV. The first model revealed a significant overall effect of different types of childhood maltreatment (CTQ subscales). A unique significant effect was found for sexual abuse, when controlling for the other types of abuse and neglect, as well as gender and age, indicating that those who reported CSA also reported more frequent sexual coercion in intimate relationships. In addition, severity of BPD features, maladaptive cognitive emotion regulation, and dissociation all positively predicted sexual violence in intimate relationships (see Table 2).

**Table 2:** Results of the linear regression analyses predicting sexual partner violence

**Model 1**	*F*	*df*	*p*	*R*^*2*^	*R*^*2*^_*adj*_
	*3.79*	*6*	*.001*	*.033*	*.024*
**Predictors**	*B*	*SE*	*p*	*CI (95%)*	
**Child Sexual Abuse**	**.508**	**.157**	**.001**	**[.199, .816]**	
Emotional Abuse	−.186	.142	.191	[−.465, .093]	
Physical Abuse	.370	.200	.064	[−.022, .762]	
Neglect	.247	.192	.199	[−.130, .624]	
Age	.011	.038	.771	[−.063, .085]	
Gender	−.096	.850	.910	[−1.765, 1.573]	
**Model 2**	*F*	*df*	*p*	*R*^*2*^	*R*^*2*^_*adj*_
	*3.637*	*3*	*.013*	*.016*	*.012*
**Predictors**	*B*	*SE*	*p*	*CI (95%)*	
**BPD features**	**.105**	**.033**	**.001**	**[.041, .169]**	
Age	.050	.038	.190	[−.025, .126]	
Gender	.110	.832	.895	[−1.524, 1.745]	
**Model 3**	*F*	*df*	*p*	*R*^*2*^	*R*^*2*^_*adj*_
	*27.625*	*3*	*<.001*	*.111*	*.107*
**Predictors**	*B*	*SE*	*p*	*CI (95%)*	
**Coping**	**.968**	**.106**	**<.001**	**[.759, 1.177]**	
Age	.022	.037	.549	[−.050, .094]	
Gender	−.390	.811	.631	[−1.982, 1.203]	
**Model 4**	*F*	*df*	*p*	*R*^*2*^	*R*^*2*^_*adj*_
	*26.956*	*3*	*<.001*	*.112*	*.108*
**Predictors**	B	SE	p	CI (95%)	
**Dissociation**	**.226**	**.025**	**<.001**	**[.176, .275]**	
Age	.069	.037	.063	[−.004, .141]	
Gender	.017	.817	.983	[−1.587, 1.621]	
**Model 5**	*F*	*df*	*p*	*R*^*2*^	*R*^*2*^_*adj*_
	*19.03*	*6*	*<.001*	.*393*	.*154*
**Predictors**	B	SE	p	CI (95%)	
**CSA**	**.444**	**.155**	**.004**	**[.139, .749]**	
BPD features	.043	.036	.235	[−.113, .028]	
**Coping**	**.636**	**.131**	**<.001**	**[.379, .893]**	
**Dissociation**	**.145**	**.033**	**<.001**	**[.079, .209]**	
Age	.031	.038	.408	[−.042, .105]	
Gender	−.489	.829	.555	[−2.116, 1.139]	

Note: *CI* 95% Bootstrapping confidence interval; *p* < .0001 ***, *p* < .01 **, *p* < .05 *

Significant effects are highlighted in bold. *BPD* Borderline Personality Disorder features as measured with the Personality Assessment Inventory Borderline Personality Features scale (mean PAI-BOR total score). Child sexual, emotional, and physical abuse, and neglect were measured with the Childhood Trauma Questionnaire (mean CTQ subscale scores); *Coping* Cognitive Emotion Regulation Questionnaire (mean CERQ subscale score), *Dissociation* Dissociative Experience Scale (mean DES total score). Age and gender are covariates

The models with maladaptive cognitive emotion regulation and dissociation respectively as predictors had the best model fit (R^2^_adj_ = .107, and R^2^_adj_ = .108). Including all predictors in one model revealed significant associations for maladaptive cognitive emotion regulation and dissociation, while CSA also remained significant (see Table 2). Age, and gender had no significant effects in none of the models.

### Mediation analysis

#### Results of the separate mediation analyses

##### BPD features

A significant indirect effect was found for BPD features (B = .077, SE = .037, CI: [.014, .160]). Higher severity of CSA predicted more BPD features (B = .957, SE = .175, t = 5.47, *p* < .0001, CI: [.614, 1.299]). More severe BPD features in turn predicted more frequent sexual IPV (B = .080, SE = .033, t = 2.41, *p* < .05, CI: [.015, .146]). The direct effect of CSA severity on sexual IPV was still significant (B = .547, SE = .154, t = 3.56, *p* < .0001, CI: [.245, .849]).

##### Dissociation

There was a significant indirect effect of CSA severity through dissociation on sexual IPV (B = .230, SE = .085, CI: [.166, .498]). CSA positively predicted dissociation (B = .1.416, SE = .231, t = 6.15, *p* < .0001, CI: [.963, 1.869]). Higher dissociation in turn predicted more sexual intimate partner violence (B = .212, SE = .026, t = 8.18, *p* < .0001, CI: [.160, .262]). The direct effect of CSA severity on sexual IPV was still significant (B = .365, SE = .155, t = 2.35, *p* < .05, CI: [.060, .670]).

##### Maladaptive cognitive emotion regulation

There was a significant indirect effect of CSA severity through maladaptive cognitive emotion regulation on sexual IPV (B = .159, SE = .066, CI: [.041, .302]). CSA positively predicted more maladaptive cognitive emotion regulation (B = .177, SE = .051, t = 3.47, *p* < .001, CI: [.077, .277). Maladaptive cognitive emotion regulation in turn predicted more sexual intimate partner violence (B = .901, SE = 106, t = 8.51, *p* < .0001, CI: [.693, 1.109]). The direct effect of CSA severity on sexual IPV was still significant (B = .431, SE = .140, t = 3.08, *p* < .01, CI: [.156, .705]).

#### Results of the multiple mediation analysis

**Fig. 1:**
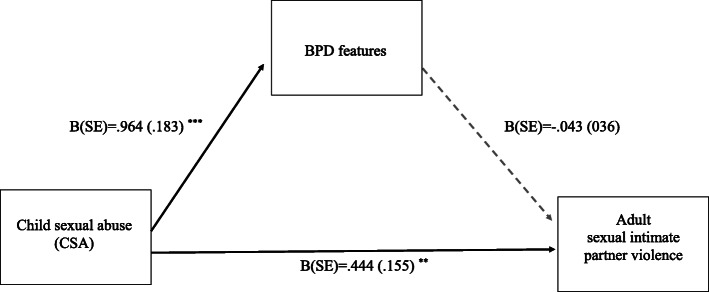
This figure shows coefficients with standard error (SE) of the multiple mediation model for Borderline Personality Disorder (BPD) features as mediator. Experiences of CSA (childhood sexual abuse) was the predictor (X) variable, frequency of sexual violence within the same adult intimate relationship was the outcome variable. This model further included dissociation and maladaptive cognitive coping as mediator variables as well as age and gender as covariates. Note: *p* < 0001 ***, *p* < 01 **

After including all mediators in one model. The direct effect of CSA severity on sexual IPV was still significant (B = .444, SE = .155, t = 2.86, *p* < .05, CI: [.139, .749]). When accounting for the effect of the other mediator variables, the indirect effect of CSA through BPD features on sexual IPV was not significant anymore (B = -.047, SE = .041, CI: [−.137, .030]). As illustrated in Fig. 1, higher severity of CSA predicted more BPD features (B = .964, SE = .183, t = 5.27, *p* < .0001, CI: [.604, 1.323]) but the association between BPD features and sexual IPV was insignificant (B = −.043, SE = .036, t = 1.19, *p* > .05, CI: [−.114, .028]).

**Fig. 2:**
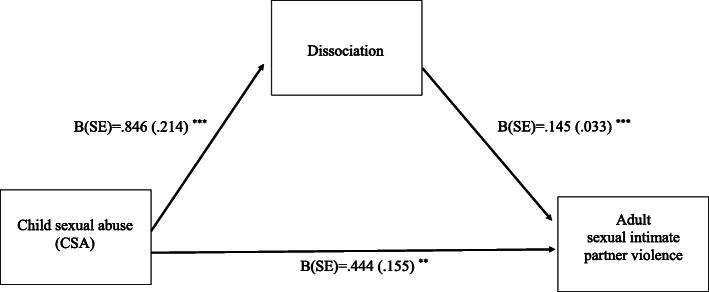
This figure shows coefficients with standard error (SE) of the multiple mediation model for dissociation as mediator. Experiences of CSA (childhood sexual abuse) was the predictor (X) variable, frequency of sexual violence within the same adult intimate relationship was the outcome variable. This model further included borderline personality disorder features and dissociation and as mediator variables as well as age and gender as covariates. Note: *p* < 0001 ***, *p* < 01 **

There was still a significant indirect effect via dissociation (B = .202, SE = .094, CI: [.057, .425]). As depicted in Fig. 2, CSA positively predicted dissociation (B = .846, SE = .214, t = 3.94, *p* < .001, CI: [.425, 1.267]), which in turn predicted more sexual IPV (B = .145, SE = .033, t = 4.38, *p* < .0001, CI: [.079, .209]).

**Fig. 3:**
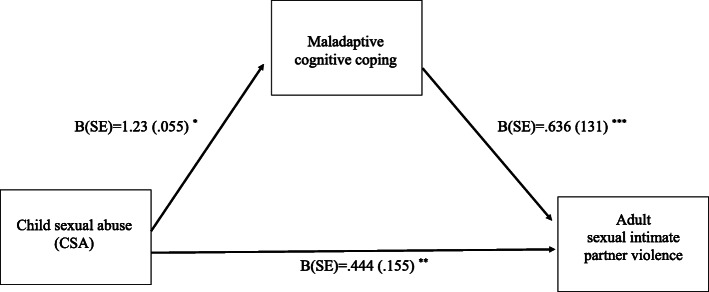
This figure shows coefficients with standard error (SE) of the multiple mediation model for maladaptive cognitive coping as mediator. Experiences of CSA (childhood sexual abuse) was the predictor (X) variable, frequency of sexual violence within the same adult intimate relationship was the outcome variable. This model further included borderline personality disorder features and maladaptive cognitive coping and as mediator variables as well as age and gender as covariates. Note: *p* < .0001 ***, *p* < .01 **, *p* < .05 *

In this multiple mediation model, a small significant indirect effect was also observed for maladaptive cognitive emotion regulation (B = .081, SE = .050, CI: [.001, .197]). As shown in Fig. 3, CSA positively predicted more maladaptive cognitive emotion regulation (B = 1.23, SE = .055, t = 2.25, *p* < .05, CI: [.016, .230]). Maladaptive cognitive emotion regulation in turn predicted more sexual intimate partner violence (B = .636, SE = .131, t = 4.86, *p* < .0001, CI: [.379, .893]).

The mediated mediation analysis revealed that the effect of BPD features was mediating by dissociation (B = .078, SE = .035, CI: [.022, .158]), but not by maladaptive emotion regulation (B = .0002, SE = .008, CI: [−.018, .016).

The analysis, which only included participants who endorsed CSA (*n* = 100) revealed similar results (see Supplemental Table 3).

## Discussion

Using self-report data from an anonymous cross-sectional online survey in *n* = 633 participants, we confirmed earlier findings of a strong positive association between childhood sexual abuse (CSA) and sexual violence in intimate adult relationships. Severity of CSA was associated with higher frequency of sexual intimate partner violence (IPV), also when accounting for other forms of childhood maltreatment. As expected, BPD features, dissociation and maladaptive cognitive emotion regulation were not only correlated to CSA but also predicted more frequent sexual IPV, mediating the association between these variables. When including the three mediator variables together in one model, only dissociation and maladaptive emotion regulation had a significant effect, while the mediating effect of BPD features was not significant anymore. A mediated mediation analysis suggests that dissociation explained the effect of CSA through BPD features (on sexual IPV).

Findings add to the existing literature on sexual re-victimization [2, 11]. Severity of childhood maltreatment (abuse and neglect) has been identified as one of the most consistent risk factors for re-victimization (1). Child sexual abuse, in particular, has been linked to re-victimization occurring in intimate relationships (2). In line with this, we found a unique predictive effect of CSA on sexual IPV, while controlling for other (emotional and physical) types of abuse and neglect.

Moreover, our findings are in line with theories proposing that specific sequelae of CSA, i.e., maladaptive emotion regulation and dissociation, underlie the increased risk of sexual re-victimization [2]. These trauma models propose that sexually abused children become more vulnerable to re-victimization due to alterations in emotional-cognitive processing and emotional regulation following CSA. Maladaptive cognitive emotion regulation strategies, such as self-blame, may prevent victims from reaching out for support and disclosing the abuse to others [25, 26], which makes it more difficult to deal with ongoing abuse [17].

Trauma models further propose that dissociation may be understood as a form of emotion modulation as it can help creating an inner distance to overwhelming experiences, e.g., during CSA [28] At the same time, dissociation interferes with cognitive information processing, e.g., perceived control, and assertiveness in abusive intimate relationships. Thereby, dissociation may increase the risk for sexual revictimization [31]. Previous prospective studies provided evidence that dissociation is a risk factor for sexual revictimization [32] and mediates the effect of CSA on IPV [33]. These findings are further supported by our cross-sectional study. Importantly, dissociation may account for the mediating effect of CSA through BPD features on sexual IPV. In the current study, BPD features had a significant mediating effect, when included as single mediator variable in a simple mediation model, i.e., without accounting for the effect of the other mediator variables. In line with this, a previous study [19] found that BPD features - especially disturbed identity and relationship problems – mediate the relation between childhood maltreatment severity and IPV frequency. Since dissociation is closely linked to disturbed identity in BPD [46, 47], it remained unclear whether part of this effect was explained by dissociation. Our current findings argue in this direction, while the current finding needs to be interpreted with caution due to our cross-sectional design. Prospective studies, which assess BPD features and dissociation (as well as CSA and sexual IPV) at different time points, are needed to confirm our preliminary results. Future studies should also assess contextual factors, e.g., the cultural or family environmental in which sexual IPV occurs [16]. IPV is often reciprocal, i.e., both partners engage in violent acts, when conflicts escalate [62]. Therefore, partner dynamics (i.e., co-occurrence of perpetration and victimization) may play an important role in this respect.

The current study focused on sexual forms of child abuse and IPV, as more research on this specific form of victimization is warranted (see) [2]. Future studies should investigate the role of BPD features and dissociation in the context of other forms of abuse / IPV. It might be in the context of overall abuse / IPV severity that BPD features come into play as a mediating factor, irrespective of the influence of dissociation. Moreover, one of our inclusion criteria was that participants were (or had previously been) in a long-term relationship. Violence in short-term relationships (e.g., dating violence) may involve different risk factors and mechanisms [34]. To better understand the exact emotional and cognitive sub-processes which play a role in sexual re-victimization, future studies may include behavioural measures (experimental emotion regulation tasks), neurobiological measures (e.g., neuroimaging, psychophysiological assessments) and electronic diaries. Future studies should also involve a broader age range and use prospective designs, since adolescents show increased rates of dating violence [34].

To our knowledge, this is the first study that investigated the mediating effect of BPD features, dissociation, and maladaptive coping in one model. Our findings need to be interpretated in the light of several limitations. While our statistical analyses were based on theoretical models and previous longitudinal studies, the cross-sectional correlational design of the current study does not allow conclusions about cause and effects of the above-mentioned factors. The investigated variables may likely act together, creating complex dynamics that are hard to break. Negative relationship experiences may reinforce maladaptive emotion regulation and dissociation, and potentially increase the severity of BPD symptoms, such as affective instability and self-harming impulsivity. In a similar vein, negative relationship experiences may increase the recall and vividness of traumatic childhood memories, making them more likely to be activated and to interfere with current life experiences. Since childhood maltreatment was assessed by self-reports and in a retrospective, subjective manner, these reports may have been colored by current mood. Although the survey was fully anonymous, participants may have provided socially desirable or distorted answers, e.g., due to limited awareness and insight or different subjective interpretations of measured concepts. Including more than one mediator variable in the model may have reduced statistical power and increased the chance of false negative findings (e.g., regarding the effect of BPD features). Moreover, individuals with more severe BPD are less likely to have long-term romantic relationships and our inclusion criteria may have limited the representativeness of our BPD sample. Further limiting the representativeness of our sample, a relatively high number of participants reported incidents of sexual IPV and scored above the cut-off for clinically relevant BPD features and dissociation. This is probably be due to the fact that we recruited at online platforms for sufferers from domestic violence. Even though we additionally recruited via other social media and research platforms of Leiden University, our recruitment led to a selective sample.

Although approximately half of the sub-sample of individuals who reported CSA presented with clinically relevant BPD features (i.e., scored above the established cut-off for this scale), our findings need to be replicated in clinical samples, as we did not include semi-structured clinical interviews that are needed to confirm a clinical diagnosis. The majority of the participants, especially in the subgroup who reported CSA, were female. Although this reflects the unequal prevalence observed in more representative samples [10], this led to an overrepresentation of women in our study. Men with BPD may be more likely to have higher levels of novelty seeking or show antisocial behaviour, whereas women may be more likely to experience anxiety and depression. Lastly, PTSD is a highly co-occurring condition in BPD [63, 64], which may have influenced our results. The recently introduced ICD 11 diagnosis of complex PTSD may provide a conceptual framework for understanding overlapping symptoms of both BPD and PTSD, such as difficulties in emotion regulation and interpersonal problems, which may underlie the increased risk of sexual IPV.

Given the detrimental effect of CSA on physical and mental health outcomes (1–9), research in this area can help to deepen the understanding of factors involved in a potential cycle of abuse.

Early psychological interventions for persons who experienced abuse in childhood may help to prevent sexual abuse in adult intimate partner relationships. Training in emotion regulation, stress coping, and interpersonal skills (e.g., assertiveness) may help survivors of CSA to identify and adjust dysfunctional attitudes towards themselves and their sexuality, breaking the pattern of sexual re-victimization. Dissociative responses should be closely monitored and targeted in treatment. A combination of trauma-focused interventions (e.g., exposure-based treatment), stabilizing interventions, and skills training (e.g., as in Dialectical Behaviour Therapy) [65] may be efficient for patients, who have experienced ongoing interpersonal violence [66].

## Conclusion

In conclusion, our findings suggest that CSA severity, BPD features, dissociation, and maladaptive emotion regulation are significant predictors of sexual IPV. Findings provide first evidence that dissociation and maladaptive cognitive emotion regulation underlie a sexual IPV in individuals who report CSA. Findings need to be corroborated by prospective studies with longitudinal repeated measure designs to identify risk factors that predict sexual re-victimization in a clear temporal sense. Ultimately, this may help developing interventions to prevent sexual re-victimization.

## Supplementary Information

**Additional file 1: Supplemental Table 1.** Demographic characteristics.

**Additional file 2: Supplemental Table 2.** Correlations in the whole sample.

**Additional file 3: Supplemental Table 3.** Results of the mediation analysis including only participants who reported CSA (*n* = 100).

## Data Availability

Data access can be requested from the corresponding author [AKU] on reasonable demand.
