# Supplementary material for: Linking experiences of child sexual abuse to adult sexual intimate partner violence: the role of borderline personality features, maladaptive cognitive emotion regulation, and dissociation
Source: Borderline Personal Disord Emot Dysregul. 2021 Apr 1;8:10. doi: 10.1186/s40479-021-00150-0 (PMC8015045; doi:10.1186/s40479-021-00150-0)
Supplement: Supplementary file 1 — Additional file 1: Supplemental Table 1. Demographic characteristics. [file 40479_2021_150_MOESM1_ESM.docx]

Supplemental Table 1

Demographic characteristics

| Demographics | Frequency n (percentages %) | | Group comparisons |
| --- | --- | --- | --- |
|  | Completers | Drop-outs |  |
| *Gender*  Female  Male  Both | *n = 633*  448 (70.8%)  185 (29.2%)  0 | *n = 336*  212 (63.1%)  123 (36.6%)  1 (0.3%) | χ² (2, 969) = 7.546, *p* < .023 |
| *Education*  Primary School  Secondary School  Bachelor Degree  Master Degree  PhD  Vocational Training  Other | *n = 633*  5 (0.8%)  343 (54.2%)  165 (26.1%)  73 (11.5%)  10 (1.6%)  29 (4.6%)  15 (2.4%) | *n = 336*  10 (3.0%)  185 (55.1%)  94 (28.0%)  17 (5.1%)  2 (0.6%)  13 (3.9%)  14 (4.2%) | χ² (6, 969) = 22.81, *p* < .001 |
| *Relationship Status*  Currently in a relationship  Married  Divorced  Separated  Widowed  Other | *n = 622*  342 (54.0%)  87 (14.0%)  2 (0.3%)  1 (0.2%)  2 (0.3%)  5 (0.8%) | *n = 335*  65 (19.4%)  13 (3.9%)  2 (0.6%)  4 (1.2%)  1 (0.3%)  1 (0.3%) | χ² (6, 957) = 189.104, *p* < .001 |
| *Nationality*  European  Asian  North American  South American  Middle East  2 nationalities  3 nationalities  Other | *n = 633*  528 (83.4%)  16 (2.5%)  5 (0.8%)  5 (0.8%)  44 (7.0%)  6 (0.9%)  7 (1.1%)  22 (3.5%) | *n = 334*  251 (75.1%)  12 (3.6%)  0 (0.0%)  7 (2.1%)  7 (2.1%)  14 (4.2%)  19 (5.7%)  24 (7.2%) | χ² (8, 967) = 66.52, *p* < .001 |
