# Supplementary material for: Linking experiences of child sexual abuse to adult sexual intimate partner violence: the role of borderline personality features, maladaptive cognitive emotion regulation, and dissociation
Source: Borderline Personal Disord Emot Dysregul. 2021 Apr 1;8:10. doi: 10.1186/s40479-021-00150-0 (PMC8015045; doi:10.1186/s40479-021-00150-0)
Supplement: Supplementary file 2 — Additional file 2: Supplemental Table 2. Correlations in the whole sample. [file 40479_2021_150_MOESM2_ESM.docx]

Supplemental Table 2

Correlations in the whole sample

|  | *CTS*  IPV | *CTQ* Sexual Abuse | *PAI-BOR* | *DES* | *CERQ* | *Age* | *Gender* |
| --- | --- | --- | --- | --- | --- | --- | --- |
| *CTS* Sexual Coercion | - |  |  |  |  |  |  |
| *CTQ* Sexual Abuse | .09 * | - |  |  |  |  |  |
| *PAI-BOR* | .11 ** | .22 *** | - |  |  |  |  |
| *DES* | .42 *** | .20 *** | .44 *** | - |  |  |  |
| *CERQ* | .51 *** | .11 ** | .34 *** | .66 *** | - |  |  |
| *Age* | .06 | .10 ** | -.10 ** | -.09 ** | -.06 | - |  |
| *Gender* | .03 | .09 ** | .07 * | .02 | .002 | -.19 *** | - |

Note: N=643 *** p<.001, ** p<.01, * p<.05

Correlations in the subsample (n=100)

|  | *CTS*  IPV | *CTQ* Sexual Abuse | *PAI-BOR* | *DES* | *CERQ* | *Age* | *Gender* |
| --- | --- | --- | --- | --- | --- | --- | --- |
| *CTS* Sexual Coercion | - |  |  |  |  |  |  |
| *CTQ* Sexual Abuse | .27 ** | - |  |  |  |  |  |
| *PAI-BOR* | .27 ** | .34 *** | - |  |  |  |  |
| *DES* | .48 *** | .33 *** | .55 *** | - |  |  |  |
| *CERQ* | .52 *** | .25 ** | .56 *** | .61 *** | - |  |  |
| *Age* | -.08 | .15 | -.24 ** | -.18 * | .17 * | - |  |
| *Gender* | .227 * | .21 ** | .08 | .039 | .02 | .001 | - |

Note: *** p<.001, ** p<.01, * p<.05
