# Supplementary material for: Linking experiences of child sexual abuse to adult sexual intimate partner violence: the role of borderline personality features, maladaptive cognitive emotion regulation, and dissociation
Source: Borderline Personal Disord Emot Dysregul. 2021 Apr 1;8:10. doi: 10.1186/s40479-021-00150-0 (PMC8015045; doi:10.1186/s40479-021-00150-0)
Supplement: Supplementary file 3 — Additional file 3: Supplemental Table 3. Results of the mediation analysis including only participants who reported CSA (n = 100). [file 40479_2021_150_MOESM3_ESM.docx]

Supplemental Table 3

Results of the mediation analysis including only participants who reported CSA (n = 100).

| *BPD features:*  No significant indirect effect was found for BPD features (B=-.051, SE=.083, CI: [-.100, .235]). Higher severity of CSA predicted more BPD features (B=.763, SE=.250, t=3.06, p<.001, CI: [.268, 1.257]) but the association between BPD features and sexual IPV was insignificant (B= -.067, SE=.126, t=0.54, p>.05, CI: [-.183, .318]). |
| --- |
| *Dissociation*:  There was a significant indirect effect of CSA severity through dissociation on sexual IPV (B=.230, SE=.160, CI: [.096, 634]). CSA positively predicted dissociation (B(SE)=.935 (.398), t=2.34, p=.021, CI: [.145, 1.725]). Higher dissociation in turn predicted more sexual intimate partner violence (B(SE)=.246 (.082), t=3.02, p<.010, CI: [.084, .407]). |
| *Maladaptive cognitive emotion regulation*:  There was a significant indirect effect of CSA severity through maladaptive cognitive emotion regulation on sexual IPV (B=.208, SE=.128, CI: [.016, .510]). CSA positively predicted more maladaptive cognitive emotion regulation (B(SE)=.204 (.077), t=2.67, p<.01, CI: [.057, 356]). Maladaptive cognitive emotion regulation in turn predicted more sexual intimate partner violence (B(SE)=1.020 (380), t= 2.69, p<.01, CI: [.266, 1.773]). |

Note: To test the robustness of our findings, we repeated the mediation analysis including only participants who reported CSA (n = 100). In this analysis, frequency of sexual abuse in intimate relationships (CTS-2 sexual coercion subscale) was defined as outcome measure (Y variable). Severity of CSA (CTQ sexual abuse subscale) was the predictor (X). BPD features (PAI-BOR total), cognitive emotion regulation (CERQ ‘maladaptive‘ subscale score), and dissociation (DES sum score) were included as mediator variables. A bootstrapping function based on 5000 samples and a confidence interval of 95% was used to quantify direct and indirect effects. Age and gender were added as covariates.
